# Supplementary figures and images for: Economic development, weather shocks and child marriage in South Asia: A machine learning approach
Source: PLoS One. 2022 Sep 1;17(9):e0271373. doi: 10.1371/journal.pone.0271373 (PMC9436147; doi:10.1371/journal.pone.0271373)

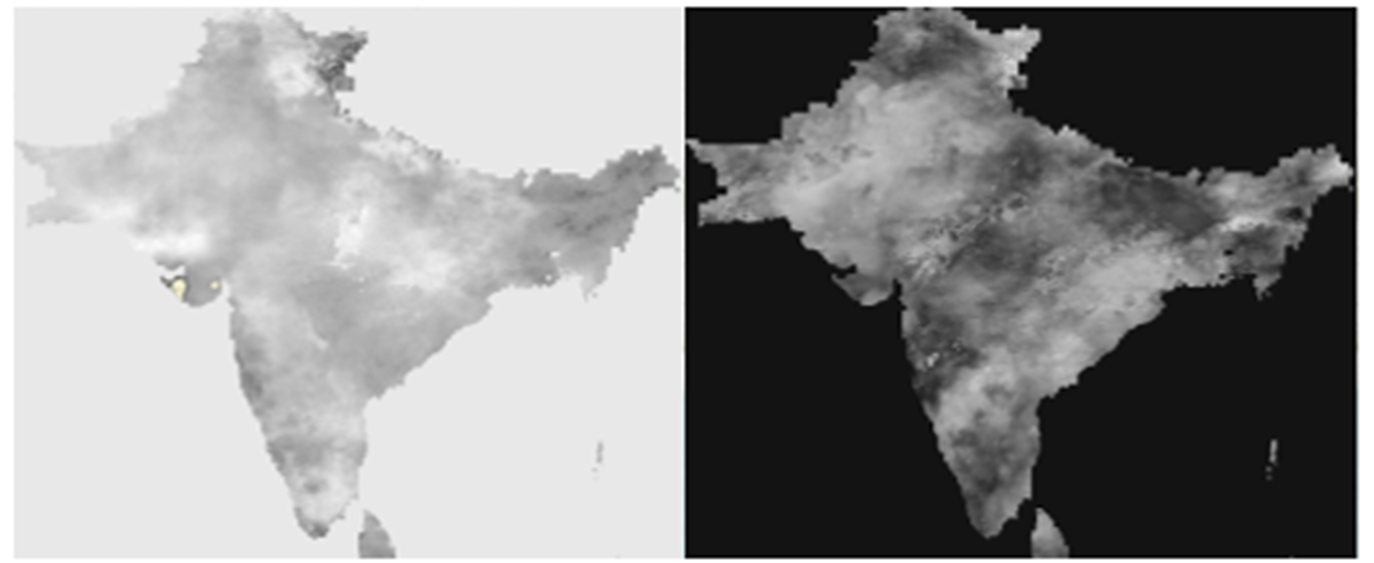

Supplement: S1 Fig — Note: Own calculations based on women age 18 to 22 at the time of interview. Outlier observations of women that were interviewed outside the standard survey period (i.e., women that were interviewed in later or earlier years than the rest of same survey wave) were removed to avoid plotting means that are based on few observations. Shaded area shows 95% confidence bands. (TIF) [file pone.0271373.s001.tif]

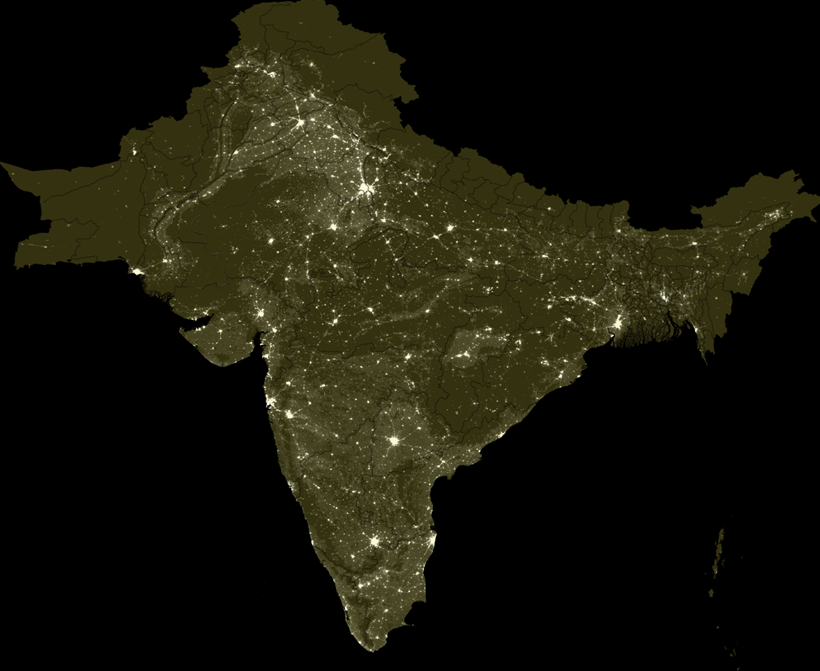

Supplement: S2 Fig — Minimum (drought indicator; left) and maximum (extremely wet conditions; right) SPEI, 2012. Note: Own calculation based on SPEI data provided by Aadhar and Mishra (2017). (TIF) [file pone.0271373.s002.tif]

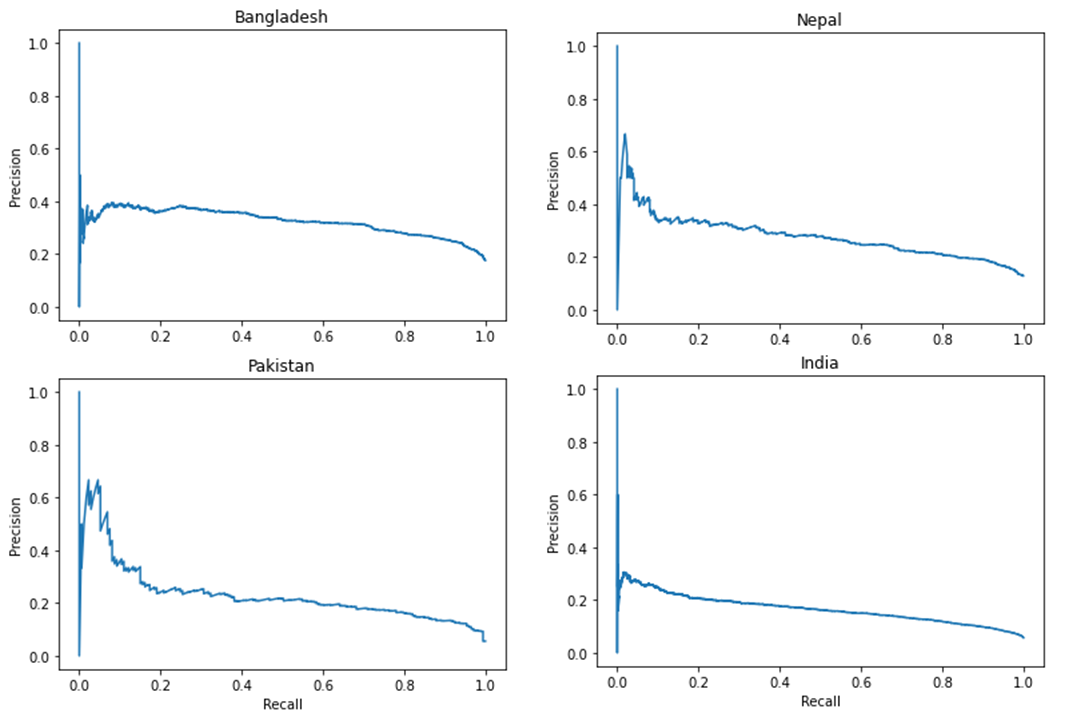

Supplement: S3 Fig — Note: Own calculation based on NOOA NTL data of 2013. (PNG) [file pone.0271373.s003.png]
