# Supplementary material for: Economic development, weather shocks and child marriage in South Asia: A machine learning approach
Source: PLoS One. 2022 Sep 1;17(9):e0271373. doi: 10.1371/journal.pone.0271373 (PMC9436147; doi:10.1371/journal.pone.0271373)
Supplement: S1 Table — (DOCX) [file pone.0271373.s005.docx]

**Table S1. Summary of Results for the 18-22 Age Bracket**

| **Results** | **Bangladesh** | **Nepal** | **Pakistan** | **India** | **All Countries** |
| --- | --- | --- | --- | --- | --- |
| **Panel A: confusion matrix** | | | | | |
| True Negative | 17870 | 2275 | 4332 | 43552 | 66696 |
| False Positive | 2430 | 1087 | 562 | 20143 | 25554 |
| False Negative | 22 | 123 | 21 | 680 | 658 |
| True Positive | 829 | 345 | 114 | 2370 | 3846 |
| **Panel B: performance metrics** | | | | | |
| ROC AUC | 0.96 | 0.77 | 0.94 | 0.80 | 0.87 |
| Accuracy | 0.88 | 0.68 | 0.88 | 0.69 | 0.73 |
| F1 | 0.40 | 0.36 | 0.28 | 0.19 | 0.23 |
| Precision | 0.25 | 0.24 | 0.17 | 0.11 | 0.13 |
| Recall | 0.97 | 0.74 | 0.84 | 0.78 | 0.85 |
