# Supplementary material for: Economic development, weather shocks and child marriage in South Asia: A machine learning approach
Source: PLoS One. 2022 Sep 1;17(9):e0271373. doi: 10.1371/journal.pone.0271373 (PMC9436147; doi:10.1371/journal.pone.0271373)
Supplement: S2 Table — (DOCX) [file pone.0271373.s006.docx]

**Table S2.** **Logistic Regression Model**

| **Results** | **Bangladesh** | **Nepal** | **Pakistan** | **India** | **All Countries** |
| --- | --- | --- | --- | --- | --- |
| **Panel A: confusion matrix** | | | | | |
| True Negative | 12444 | 2973 | 4586 | 63804 | 83820 |
| False Positive | 8 | 18 | 0 | 0 | 11 |
| False Negative | 731 | 404 | 173 | 3598 | 4913 |
| True Positive | 9 | 9 | 0 | 0 | 12 |
| **Panel B: performance metrics** | | | | | |
| ROC AUC | 0.79 | 0.76 | 0.73 | 0.79 | 0.79 |
| Accuracy | 0.94 | 0.88 | 0.96 | 0.95 | 0.94 |
| F1 | 0.02 | 0.04 | 0 | 0 | 0.00 |
| Precision | 0.53 | 0.33 | 0 | 0 | 0.52 |
| Recall | 0.01 | 0.02 | 0 | 0 | 0.00 |
| ***Note****: Panel A reports on count of cases in the test data (20% of full sample) and Panel B reports shares.* | | | | | |
