# Supplementary material for: Economic development, weather shocks and child marriage in South Asia: A machine learning approach
Source: PLoS One. 2022 Sep 1;17(9):e0271373. doi: 10.1371/journal.pone.0271373 (PMC9436147; doi:10.1371/journal.pone.0271373)
Supplement: S3 Table — (DOCX) [file pone.0271373.s007.docx]

**Table S3.** **Results with spatially blocked partitioning**

| **Results** | **Bangladesh** | **Nepal** | **Pakistan** | **India** | **All Countries** |
| --- | --- | --- | --- | --- | --- |
| **Panel A: confusion matrix** | | | | | |
| True Negative | 9698 | 1235 | 3479 | 43283 | 64160 |
| False Positive | 1728 | 1847 | 371 | 19834 | 19957 |
| False Negative | 123 | 37 | 47 | 765 | 1300 |
| True Positive | 650 | 437 | 104 | 2706 | 3653 |
| **Panel B: performance metrics** | | | | | |
| ROC AUC | 0.91 | 0.77 | 0.91 | 0.80 | 0.83 |
| Accuracy | 0.85 | 0.47 | 0.90 | 0.69 | 0.76 |
| F1 | 0.41 | 0.32 | 0.33 | 0.21 | 0.26 |
| Precision | 0.27 | 0.19 | 0.22 | 0.12 | 0.15 |
| Recall | 0.84 | 0.92 | 0.69 | 0.78 | 0.74 |
| ***Note****: Panel A reports on count of cases in the test data (20% of full sample) and Panel B reports shares. Compared to the main results, for these results spatially blocked partitioning (72) was used to ensure that test data only included survey clusters that are not part of the training data.* | | | | | |
