# Supplementary material for: Economic development, weather shocks and child marriage in South Asia: A machine learning approach
Source: PLoS One. 2022 Sep 1;17(9):e0271373. doi: 10.1371/journal.pone.0271373 (PMC9436147; doi:10.1371/journal.pone.0271373)
Supplement: S4 Table — (DOCX) [file pone.0271373.s008.docx]

**Table S4. Summary of results (with Area Under Precision-Recall Curve as performance metric)**

| **Results** | **Bangladesh** | **Nepal** | **Pakistan** | **India** | **All Countries** |
| --- | --- | --- | --- | --- | --- |
| **Panel A: confusion matrix** | | | | | |
| True Negative | 10643 | 1472 | 4460 | 43789 | 62024 |
| False Positive | 2124 | 1519 | 628 | 20015 | 22623 |
| False Negative | 67 | 53 | 45 | 798 | 1113 |
| True Positive | 673 | 360 | 128 | 2800 | 3812 |
| **Panel B: performance metrics** | | | | | |
| AUPRC | 0.32 | 0.28 | 0.24 | 0.16 | 0.21 |
| ROC AUC | 0.93 | 0.76 | 0.92 | 0.80 | 0.83 |
| Accuracy | 0.84 | 0.54 | 0.87 | 0.69 | 0.74 |
| F1 | 0.38 | 0.31 | 0.28 | 0.21 | 0.24 |
| Precision | 0.24 | 0.19 | 0.17 | 0.12 | 0.14 |
| Recall | 0.91 | 0.87 | 0.74 | 0.78 | 0.77 |
| ***Note****: Panel A reports on count of cases in the test data (20% of full sample) and Panel B reports shares.* | | | | | |
